# A New Cure for Nervous Affections

**Published:** 1847-10

**Authors:** 


*A new cure for Nervous Affections.*—M. Pallas considers that the cause of a great number of "nervous affections" is to be found in the excessive influence of atmospheric or terrestrial electricity. He states that, by adapting to bedsteads glass feet, and isolating them at about eighteen inches from the wall of the apartment, he has cured the patients sleeping upon them of a host of nervous affections.

The impression produced on the mind has probably as powerful an influence in the cure as the insulation on glass.—*Ibid.*

